# Tidal Volume Monitoring via Surface Motions of the Upper Body—A Pilot Study of an Artificial Intelligence Approach

**DOI:** 10.3390/s25082401

**Published:** 2025-04-10

**Authors:** Bernhard Laufer, Tamer Abdulbaki Alshirbaji, Paul David Docherty, Nour Aldeen Jalal, Sabine Krueger-Ziolek, Knut Moeller

**Affiliations:** 1 Institute of Technical Medicine (ITeM), Furtwangen University, 78056 Villingen-Schwenningen, Germany; tamer.abdulbakialshirbaji@hs-furtwangen.de (T.A.A.); sabine.krueger-ziolek@hs-furtwangen.de (S.K.-Z.); knut.moeller@hs-furtwangen.de (K.M.); 2 Innovation Center Computer Assisted Surgery (ICCAS), University of Leipzig, 04103 Leipzig, Germany; nour-jalal@hotmail.com; 3 Department of Mechanical Engineering, University of Canterbury, Christchurch 8140, New Zealand; paul.docherty@canterbury.ac.nz

**Keywords:** wearables, smart clothing, respiratory parameters, artificial intelligence, neuronal networks

## Abstract

The measurement of tidal volumes via respiratory-induced surface movements of the upper body has been an objective in medical diagnostics for decades, but a real breakthrough has not yet been achieved. The improvement of measurement technology through new, improved sensor systems and the use of artificial intelligence have given this field of research a new dynamic in recent years and opened up new possibilities. Based on the measurement from a motion capture system, the respiration-induced surface motions of 16 test subjects were examined, and specific motion parameters were calculated. Subsequently, linear regression and a tailored convolutional neural network (CNN) were used to determine tidal volumes from an optimal set of motion parameters. The results showed that the linear regression approach, after individual calibration, could be used in clinical applications for 13/16 subjects (mean absolute error < 150 mL), while the CNN approach achieved this accuracy in 5/16 subjects. Here, the individual subject-specific calibration provides significant advantages for the linear regression approach compared to the CNN, which does not require calibration. A larger dataset may allow for greater confidence in the outcomes of the CNN approach. A CNN model trained on a larger dataset would improve performance and may enable clinical use. However, the database of 16 subjects only allows for low-risk use in home care or sports. The CNN approach can currently be used to monitor respiration in home care or competitive sports, while it has the potential to be used in clinical applications if based on a larger dataset that could be gradually built up. Thus, a CNN could provide tidal volumes, the missing parameter in vital signs monitoring, without calibration.

## 1. Introduction

The use of artificial intelligence (AI) has proven beneficial in almost all areas of science and research in recent years. AI has opened up new perspectives in many areas of medicine, particularly in medical diagnostics. This includes the recognition of surgical phases during complex surgical workflows [1], smoke detection in minimally invasive procedures [2], the classification of surgical tools [3,4], and the automatic analysis of X-ray images [5,6]. The increasing complexity of surgical procedures leads to a high information density for physicians. With advances in computer vision, data science, and AI, it is likely that AI support will become a critical feature of future operating theatres [7,8,9]. AI-based methods in medicine have shown great potential to support doctors and medical professionals in this area and beyond [10].

AI has proven beneficial to respiratory medicine and has the potential to significantly improve the care of respiratory patients [11]. Doctors can be supported in diagnosis, and treatments can be more targeted and customised to the individual patient. Al-Anazi et al. [12] have recently shown that in addition to automatically analysing X-ray images, AI can also improve airway treatment and monitoring. AI is now also being used in the diagnosis of asthma and COPD [13]; as there are only minor differences between the two diseases in terms of clinical presentation, AI can support medical staff in making a reliable diagnosis.

Neural networks (NNs) of different architectures have been used for this purpose. In addition to simple NNs, convolutional neural networks (CNNs) and neural networks with long short-term memory (LSTM) architectures have been combined, and different pipelines have been tailored for different applications, allowing for spatio-temporal modelling. These architectures offer considerable advantages over simple NNs, especially for more specialised tasks such as the recognition of surgical tools and the associated recognition of surgical phases.

Despite the potential benefits of AI in medicine, it remains controversial, especially in the sensitive area of influencing doctors and diagnoses [14]. However, if AI is used in a targeted manner and its objectivity is guaranteed, it can help identify features and recognise new aspects, especially in large datasets or complex, uncommon presentations. The measurement of tidal volumes via respiratory-induced surface movements of the upper body has been a long-standing objective in medical diagnostics and has been included in research for decades. Konno and Mead were pioneers in this area in the 1960s [15]. Unfortunately, a breakthrough success has not yet been achieved, and only two measurement methods, optoelectronic plethysmography (OEP) [16,17] and respiratory inductance plethysmography (RIP) [18,19], have occasionally been used in clinical practice or home monitoring. OEP uses an optical motion capture system (MoCap) to determine respiratory parameters from the surface movements of the upper body, while RIP uses inductively measured changes in the cross-sectional area of the upper body. Since OEP is associated with high acquisition costs and the measurement accuracy of RIP is not sufficient for many applications [20], these two methods are used rather sporadically, and the measurement of respiratory parameters is usually based on airflow measurement through the mouth and nose using spirometers [21,22] or body plethysmographs [23,24]. Although laminar airflow can be measured accurately, the face masks or mouthpieces required for flow measurement can be uncomfortable for patients, especially during long-term measurements, and may even impact the measurement results themselves [25,26].

The measurement of respiratory volumes via surface movements of the upper body does not have these disadvantages; therefore, numerous studies have attempted to determine respiratory volumes based on different sensors and measurement techniques. Some studies analysed the respiratory-induced upper body movements [27,28,29,30], while others examined the required number of selected sensors and their positioning in a smart shirt [31,32,33,34]. Additionally, other studies used inertial measurement units (IMUs) [35,36], other belt systems, or strain gauges [37,38,39].

Improved measurement technology, the miniaturisation of sensors, and the development of new and improved sensors and sensor systems in recent years have introduced new dynamics to this field of research and opened up new opportunities. Smart shirts and intelligent garments are increasingly used in medical diagnostics and therapy monitoring [40]. However, due to the lack of accuracy in determining respiratory volume, respiratory monitoring usually only analyses the respiratory rate [36,41,42,43], which can be monitored with an adequate level of accuracy for clinical purposes.

The lack of accuracy is due to the fact that breathing has patient-specific mechanics. There are two different breathing styles in human respiration: abdominal breathing and thoracic breathing. In abdominal breathing, the change in volume is mainly apparent in the abdominal region, and the work of breathing is mainly carried out by the diaphragm; in thoracic breathing, the chest expands, which causes the necessary change in volume. In thoracic breathing, several muscle groups are involved, such as the intercostal muscles, which are responsible for the work of breathing.

In general, the distribution of abdominal and thoracic breathing varies from person to person [44,45]. Some people tend toward thoracic breathing, whereas others tend toward abdominal breathing. For this reason, the mathematical models must be adapted to the individual breathing style of the person being examined by calibrating the measuring device.

Remote monitoring of vital parameters is a future-oriented goal in medical diagnostics. In particular, the monitoring of elderly people in home care can relieve the burden on healthcare systems and enable them to live longer in their familiar environment. Additionally, remote monitoring provides a better overview of the patient’s state of health for long-term measurements.

Electrocardiogram (ECG) monitoring systems (which track heart rate and heart rate variability) have existed for some time and are suitable for remote monitoring. However, when respiratory parameters need to be included, the respiratory rate is usually monitored due to measurement simplicity. However, the reliable monitoring of respiration requires the combination of both respiratory rate and tidal volume.

One system that aims to measure respiratory volume is the Hexoskin Shirt (Montreal, QC, Canada) [46]. Unfortunately, some studies have demonstrated that the Hexoskin Shirt is only capable of determining the respiratory rate within the clinically relevant range [47,48,49]. Regarding respiratory volumes (i.e., respiratory minute volume), the Hexoskin Shirt showed large deviations—especially during physical activities. In summary, some of the new innovations are promising, but the accurate measurement of tidal volume is still far off.

This study investigates whether it is possible to estimate tidal volumes using an AI method and a linear regression method based on movement parameters from the upper body. The AI method could even enable calibration-free measurement, which would represent a significant advance in respiratory monitoring.

## 2. Materials and Methods

### 2.1. Measurement Setup

**Figure 1:**
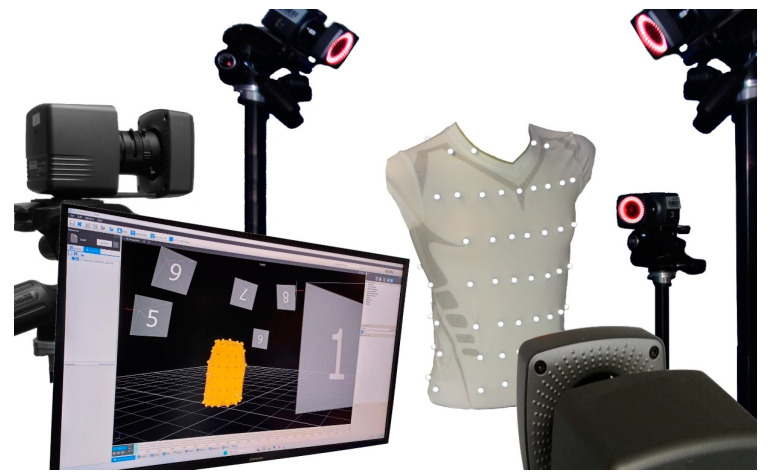
Schematic sketch of the MoCap system, using a compression shirt with 102 reflective MoCap markers.

This study utilised the methods described by Laufer et al. [27], Measurements were conducted using a MoCap system (Bonita, VICON, Denver, CO, USA) with nine infrared cameras (VICON Bonita B10, firmware version 404) to record breathing-induced upper body movements. The subjects wore a compression shirt with 102 highly reflective motion capture markers and performed specific breathing manoeuvres while surrounded by the MoCap cameras (schematic setup in Figure 1). The markers on the compression shirt were arranged at 8 different levels: 48 MoCap markers were located ventrally, 18 were located laterally, and 36 were located dorsally. Since the cervical spine is minimally affected by respiratory movements, three markers along the spine were positioned to eliminate non-respiratory movements.

Simultaneously with the MoCap system measurement, the subjects breathed through a spirometer (SpiroScout and LFX Software 1.8, Ganshorn Medizin Electronic GmbH, Niederlauer, Germany) for reference purposes.

The positions of all markers at each time point of the measurement were obtained using the MoCap system. The VICON Nexus software (version 1.8.5.6 1009h, Vicon Motion Systems Ltd.) reconstructed the positions and transferred them to MATLAB (R2024a, The MathWorks, Natick, MA, USA) for subsequent calculations. The sampling frequency of the MoCap system was set to 40 Hz, and the spirometer data (airflow and volume) were recorded at a sampling frequency of 200 Hz.

Data were obtained from seated subjects. Movements of the upper body not induced by respiration, such as bending or turning the upper body, were reduced by attaching the spirometer’s mouthpiece to a rigid holder at the level of the subject’s mouth. This setup ensured that the subjects barely moved their heads or upper bodies, thereby limiting the recorded data to respiration-related movements. Additionally, a nose clip ensured that only mouth breathing occurred.

For more details on the data acquisition, please refer to Laufer et al. [27]. In subsequent studies using these data [31,33], the optimal sensors and sensor positions were selected using regression methods. Two regression methods were evaluated, and the lasso method was selected to ensure robust determination and sparse solutions. In this way, the optimum sensor positions were determined, which allowed for the best possible determination of the tidal volumes—this study is based on these selected sensor positions.

### 2.2. Participants and Respiratory Manoeuvres

Three women and thirteen men voluntarily participated in the data acquisition. Details of the subjects are provided in Table 1. The available dataset contained only 16 subjects. Large datasets are important for research in the field of AI because they allow the AI system to learn all characteristics and features of the datasets. Therefore, due to the limitation of having only 16 subjects, a preliminary study was carried out to find out whether AI can be used for tidal volume detection.

The measurements were conducted in accordance with the tenets of the Helsinki Declaration, and ethical approval was obtained from the Human Ethics Committee of the University of Canterbury (HEC 2019/01/LR-PS) and the Ethikkommission of Furtwangen University. On average, the subjects were 25.7 ± 2.2 years old, the average weight was 69.4 ± 2.0 kg, and the average height was 1.76 ± 0.02 m.

**Figure 2:**
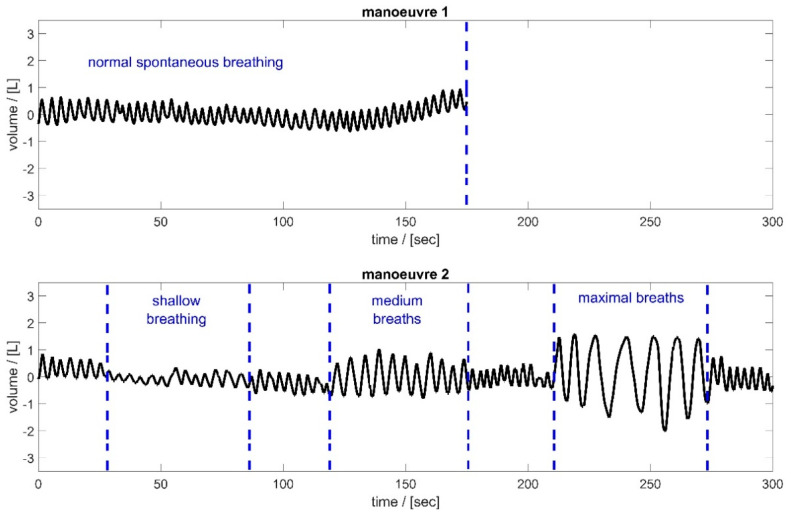
Spirometer volume data of the two respiratory manoeuvres, illustrated based on subject 2. Manoeuvre 1 (**top**): Normal spontaneous breathing for approximately 3 min; Manoeuvre 2 (**bottom**): Breathing of different tidal volumes—periods of shallow, medium, and maximal breaths between short periods of normal spontaneous breathing.

Figure 2 shows the breathing manoeuvres performed by the subjects. In the first manoeuvre, the subjects breathed normally through the spirometer for 180 s (normal spontaneous breathing). During this time, the subjects were able to familiarise themselves with the procedure. Subsequently, after a short break, the subjects were instructed in manoeuvre 2 to breathe at different tidal volumes. The goal was to cover the entire breathing spectrum—from shallow breathing to maximum breathing. The subjects breathed shallowly for 60 s, then took medium breaths and maximum breaths, each for about 60 s. These different breathing styles were interrupted by periods of normal spontaneous breathing to allow the test subjects to recover.

### 2.3. Motion Parameter Calculation—Sensors and Sensor Locations

This study was based on the movements of the 102 MoCap markers during the breathing manoeuvres shown in Figure 2. Additionally, the movement of the markers allowed for the calculation of changes in distances between neighbouring MoCap markers, changes in the circumference of the upper body at different heights, spatial displacements, and changes in tilt angles. These parameters were calculated in MATLAB (R2024b, The MathWorks, Natick, MA, USA).

**Figure 3:**
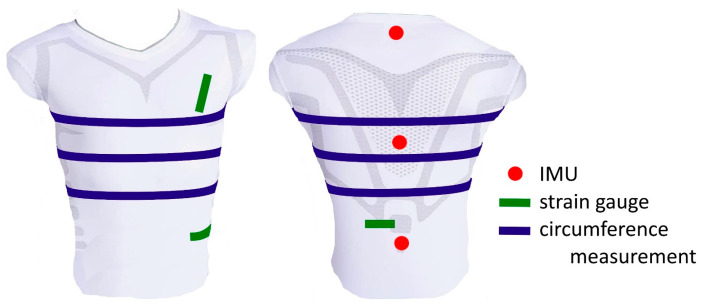
Employed distance changes (green), circumferences (blue), and IMU measurements (red) obtained via the measurement using the MoCap system and used for further analysis (ventral view (**left**) and dorsal view (**right**)).

It was observed that upper body circumferences and certain distance changes carry most of the respiratory information [33,50]. Changes in circumference can be measured using belt systems [51] or, like changes in distance, using textile strain gauges [37,39]. However, according to [33], only three of these distances and three changes in the circumference of the upper body were used for further analysis (Figure 3).

In order to correct errors caused by non-respiratory-induced movements, the spatial displacement data from three IMUs placed along the spine were used (Figure 3). Trends and offsets of all parameters were eliminated using MATLAB’s detrend function. For more details on the parameter calculation, please refer to Laufer et al. [27].

### 2.4. Data Processing and AI Design

After calculating the desired movement parameters (Figure 3—changes in circumferences, changes in MoCap marker distances, and spatial displacements), the tidal volume was estimated using two different approaches. The first approach was a regression approach, in which the second manoeuvre of the measurement (different tidal volumes) was used for individual calibration of the regression model. Thereby, the linear weighting of the employed movement parameters was determined to achieve the best possible tidal volume prediction. This weighting was then applied to the movement parameters of normal spontaneous breathing (the first manoeuvre of the measurement) to estimate the resulting tidal volumes, *V_T,Reg_*, of the individual breaths.

In the second approach, a deep learning approach based on a one-dimensional CNN network was employed. The network processes sequential input data consisting of twelve motion parameters for each individual breath. The network architecture was optimised using Bayesian optimisation to determine the best hyperparameters. The target data for the CNN were the corresponding tidal volumes obtained from the spirometer, *V_T,Spiro_*, of the corresponding breath.

**Figure 4:**
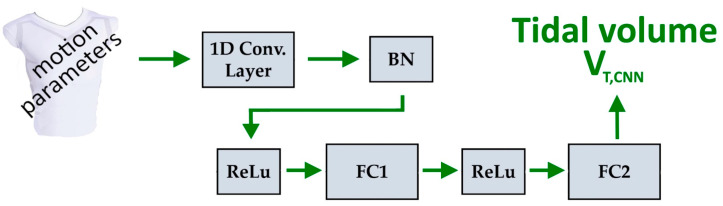
Architecture of convolutional model for estimating tidal volumes, *V_T,CNN_*. A 1D convolutional layer, a BN layer, two ReLus, and two FC layers.

The network is composed of a 1D convolutional layer and two fully connected (FC) layers. The 1D convolutional layer captures dependencies and local patterns in the input motion parameters along each breathing cycle. The input to this convolutional layer is processed by a sequence input layer that handles the time-series nature of the data. The features learned by the convolutional layer are passed through a series of two FC layers. The first FC layer learns a number of features, denoted as *feature_num*, which are then mapped to the target output in the second FC layer. To improve training stability and accelerate convergence, batch normalization (BN) and a rectified linear unit (ReLu) activation function are applied. The BN layer is used to normalise the activations of the convolutional layer. The non-linear activation function ReLu is employed on the input of each FC layer to prevent vanishing gradients and improve the network’s ability to learn complex patterns and relationships in the data. Figure 4 illustrates the model architecture.

The network was trained using the Adam optimiser, with root mean squared error (RMSE) as the loss function. L2 regularisation was applied to improve generalisability. The training parameters, such as the number of training epochs, mini-batch size, and initial learning rate, were determined through hyperparameter tuning. The dataset was shuffled at the beginning of each epoch to enhance robustness.

The hyperparameters of the proposed network were optimised using Bayesian optimisation. The optimisation process fine-tuned key parameters, including the number of training epochs (*epoch_num*), mini-batch size (*mini_bs)*, learning rate (*lr*), L2 regularisation coefficient (*L2_coe)*, convolution filter size (*filter_size)*, number of filters (*filters_num)*, and the number of neurons (*feature_num*) in the first fully connected layer (FC1). The Bayesian optimisation algorithm iteratively refined these parameters by minimising the validation error, with a maximum of 150 evaluations. The error was measured using the RMSE between the estimated and reference values. The hyperparameter values that minimised the RMSE were selected, and the final model was trained using these optimised hyperparameters (Table 2).

The neural network was trained using data from the second manoeuvre of the measurement, in which the subjects were instructed to breathe with different tidal volumes, and it was evaluated on the first manoeuvre data (normal spontaneous breathing). Finally, the resulting tidal volumes of the first manoeuvre, *V_T,CNN_*_,_ were compared with tidal volumes obtained by the spirometer, *V_T,Spiro_*, and the tidal volumes of the regression approach, *V_T,Reg_*.

## 3. Results

**Figure 5:**
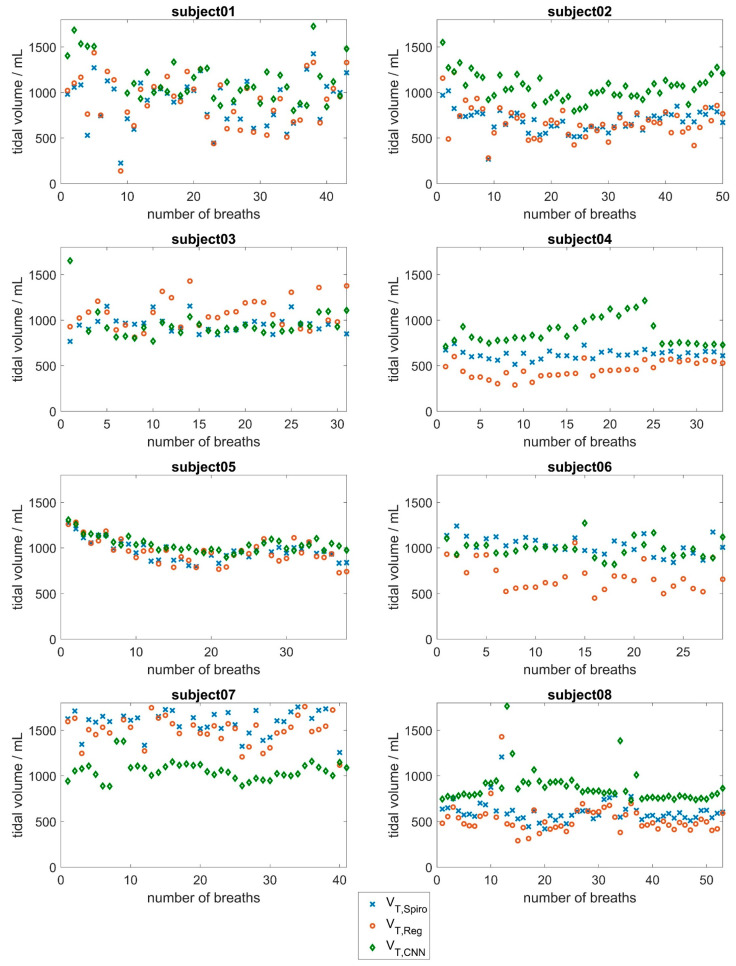
Evaluation results of the CNN approach based on surface movements for subjects 1 to 8. Tidal volumes determined by the spirometer (blue) compared to the tidal volumes determined by the CNN (red) and the tidal volumes obtained via the linear regression approach (green).

**Figure 6:**
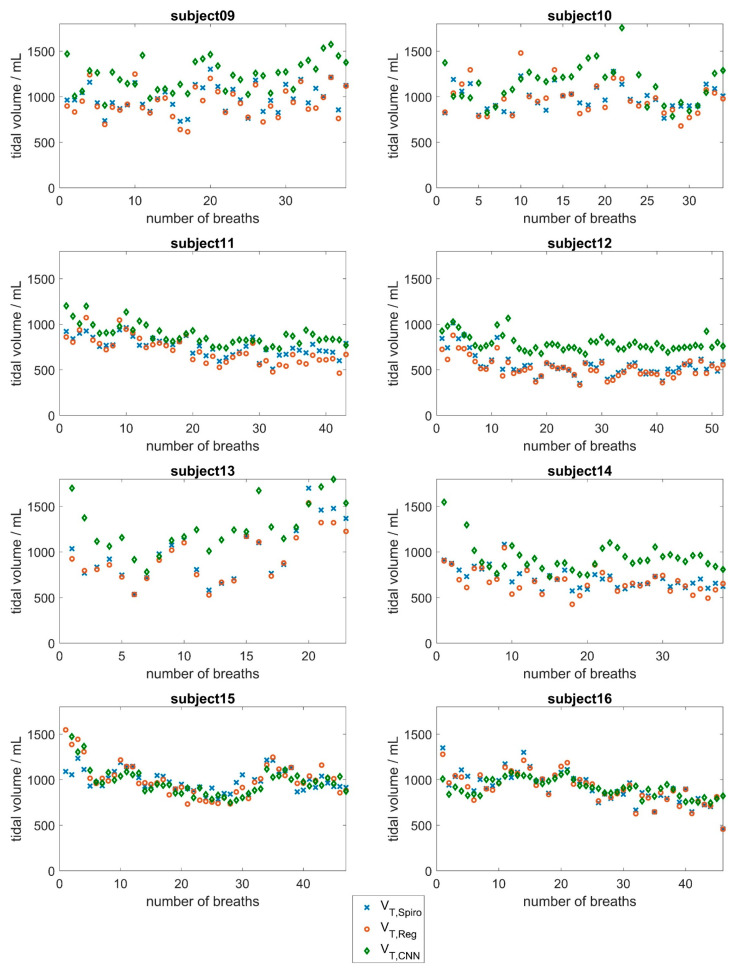
Evaluation results of the CNN approach based on surface movements for subjects 9 to 16. Tidal volumes determined by the spirometer (blue) compared to the tidal volumes determined by the CNN (red) and the tidal volumes obtained via the linear regression approach (green).

Figure 5 and Figure 6 show the tidal volume of the spirometer, *V_T,Spiro_*, compared to the tidal volumes obtained by the CNN (*V_T,CNN_*) and the tidal volumes obtained via regression, *V_T,Reg_*, for all 16 subjects. In addition, the mean absolute errors and the relative error were calculated for each subject and each approach indicated in Table 3. Please note that depending on the respiratory rate, the subjects took different numbers of breaths during the measurement period.

## 4. Discussion

Smart shirts (as well as other wearable technologies) are increasingly being integrated into medical practice, primarily for monitoring purposes [40]. Reliable measurement results for heart rate and respiratory rate have been reported [48,49]; however, in terms of respiratory volumes, the accuracies are still far from the clinically accepted range. As the reliable monitoring of respiration can only be ensured by the respiratory rate in combination with tidal volumes, the determination of tidal volumes is crucial. However, currently, tidal volumes can only be determined with sufficient accuracy for clinical application under certain conditions. A system that determines respiratory tidal volumes via surface motions of the upper body would be convenient and advantageous, especially in the case of long-term measurements, which would allow for reliable respiratory monitoring. In addition, the results would not be disturbed by the measurement itself, as is the case during flow measurements in spirometry or body plethysmography [25,26].

In this study, data from a MoCap system were utilised to measure respiration-induced movement in terms of currently available sensors for upper body movement analysis. The accuracy of the MoCap system used was in the submillimeter range and can be regarded as the gold standard for movement analysis. To ensure the best possible measurements from the MoCap system, the spirometer was fixed on a stable mount. Thus, the subjects breathing through the spirometer could only move their heads and torsos to a limited extent, and the measured surface motions were almost entirely reduced to respiration-induced movements. In addition, the subjects were able to place their hands on this mount, which significantly improved comfort during the measurement and prevented the MoCap markers from being hidden by the arms.

The various breathing manoeuvres (Figure 2) ensured that a broad range of breathing behaviours was covered. Part 1 of the measurement focused on normal breathing, which is the main application of the system. In part 2 of the measurement, the breathing spectrum ranged from shallow breathing (as in the case of people with lung disease) to maximum breathing (such as breathing activity during extensive exercise).

In 1965, Agostoni et al. showed that upper body circumferences provide a significant portion of respiratory information [50]. Laufer et al. [27] have confirmed this finding recently. The highest correlations of the analysed respiratory parameters with spirometer volume were obtained for the mean upper body circumferences in the region of the thoracic vertebra T4 and the lumbar vertebra L1. In addition, there were high correlations of local distance changes between markers in the lower dorsal region and laterally on the upper body. For this reason, the motion parameters addressed were selected for this study (Figure 3). These parameters, which can be measured using belt systems, IMUs, or strain gauges, are provided in this study to a CNN approach and a linear regression approach to predict tidal volumes.

The regression approach shows that the relative error of tidal volume determination in 11 of the 16 subjects is less than 10% of the average tidal volume breathed. This finding could allow for clinical use in these subjects. Deviations were larger in some subjects (subjects 3, 4, 6, and 8), resulting from a systematic overestimation or underestimation of the tidal volume values. Individual calibration should be checked in these cases.

With regard to the CNN approach, the results in Figure 5 and Figure 6 show good agreement between the respiratory volumes determined for subjects 5, 6, 11, 15, and 16 (mean deviations of less than 150 mL). During the measurement, it was evident that a few subjects tended to breathe abdominally, while most subjects tended to breathe thoracically. Larger deviations were observed in subjects 1, 2, 13, and 14, and especially in subject 7, with a mean deviation of about 580 mL. This evaluation underlines the fact that breathing is a highly individual process that depends on various factors, such as the age-dependent flexibility of the chest and individual distribution of chest and abdominal breathing [44,45]. At 53 years of age, subject 7 was significantly older than the other subjects, who had a mean age of 24 years. In the older subject, there may be slight deviations in the breathing movements of the upper body due to reduced flexibility of the ribs and the other bones of the thorax. Additionally, the mean tidal volume of subject 7 was significantly larger than the mean tidal volumes of the other subjects.

As most subjects in the dataset were young, lung-healthy men who tended to perform chest breathing, the CNN did not have enough training data to learn about the influence of abdominal versus chest breathing, age, or gender. Therefore, higher deviations were observed in women and in subjects whose breathing style differed from the norm (subjects 2, 4, and 14).

There is a key aspect to consider. Individual calibration is required for the linear regression approach. The measurement system must be calibrated before the measurement, under professional supervision, using a spirometer. For this purpose, subjects/patients have to visit a doctor’s office or a hospital equipped with a spirometer. This individual calibration for each subject/patient provides considerable advantages for the regression approach when comparing the performance of the two approaches, as via calibration, the underlying model is adapted directly to the individual breathing style of the subject.

On the other hand, no calibration is required for the CNN approach, resulting in significant benefits for patients and cost savings for the healthcare system. At the moment, average deviations of more than 240 mL indicate that the system is not yet sufficiently accurate for clinical use. However, this level of accuracy could allow the system to be used in home care or for analysing respiratory function in competitive sports, where accuracy is not as important as in clinical applications.

The available dataset was limited to 16 subjects. It can be assumed that the performance of the CNN will be improved if a larger dataset is used for training, as AI benefits from a large amount of data [52]. A dataset should be collected from a larger number of subjects with different body shapes, ages, genders, breathing styles, and pathologies to allow the CNN to learn the influence of these factors. This would allow for deeper insight into the breathing mechanics implied by the measured thoracic surface motions. Such a dataset could be built up gradually, and the accuracy of the CNN-based system would be improved over time. The dataset used only allows for a limited analysis and gives an indication that a CNN is certainly capable of being used in the field of respiratory monitoring. With a larger dataset, a more complex and meaningful evaluation of the results would be possible.

In addition, the sensor/parameter selection (three changes in circumferences, three changes in MoCap marker distances, and three spatial displacements) should also be reviewed using a larger dataset, as other or additional sensors could significantly enhance the performance of the measurement system. In addition to the spatial position changes in the IMU, the tilt angles and accelerations could also be used. These data are recorded by the IMU by default and could improve the performance of the system. Only data from incremental measurements were used (changes in circumferences, distances, and spatial positions). Some sensors are limited to incremental measurements (e.g., measuring the change in circumference with belt systems), as absolute values can only be measured after additional calibration.

**Figure 7:**
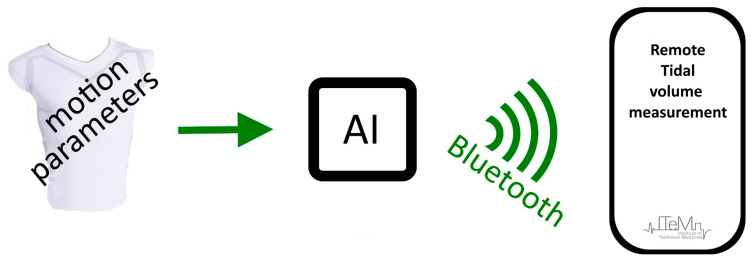
Outlook: Remote respiratory monitoring system to access tidal volume (V_T_) via motion parameters.

This study is a feasibility study in which the sensor data are derived from measurements taken by the MoCap system. There are no restrictions on measuring the respective parameters with the corresponding sensors, and it can be assumed that this can be implemented in the next step. The hardware could send the resulting tidal volume data in real time via Bluetooth from the measurement system to an app on a cell phone, as shown in Figure 7.

Improvements to the CNN approach could be achieved with other NN architectures. However, the most important aspect for better performance of the CNN method is definitely the stepwise build-up of a larger dataset. Additionally, this study was conducted only with seated subjects; the influence of posture must be investigated in a separate study, as body position could also have a significant influence on the performance of the CNN.

Further studies could show the applicability and specific requirements for people with lung disease, e.g., patients with chronic obstructive pulmonary disease or cystic fibrosis. Special care must be taken to ensure that the breathing of people with lung disease is impaired as little as possible during the measurement and that a comfortable measurement is possible. The system presented could offer significant advantages in this context, as it would enable convenient long-term monitoring for patients with lung disease.

## 5. Conclusions

The convolutional neural network approach presented in this study provides tidal volumes based on the surface movements of the upper body, which can be used for respiratory monitoring in the homecare sector or in competitive sports. The determination of tidal volumes from the surface movements of the upper body with sufficient accuracy was previously possible only under special conditions. The major advantage of this method is that no expensive calibration is required. By using a larger dataset, which could be gradually built up, it is likely that the CNN method has potential clinical applications in ventilation monitoring.

The linear regression method, which required a calibration routine, provided tidal volumes that could justify clinical use. However, this a priori calibration would be difficult to undertake in the typical workflow of a doctor’s office or hospital.

Both approaches presented here have the potential to improve the monitoring of vital signs by enabling the integration of tidal volumes into the monitoring process.

## Figures and Tables

**Table 1:** Details of the participants. Table was previously published in Laufer et al. [27].

Subject	Height [m]	Weight [kg]	BMI [kg/m^2^]	Age [years]	Gender
1	1.84	75	22.15	18	male
2	1.72	65	21.97	19	female
3	1.70	56	19.38	26	male
4	1.67	57	20.44	18	female
5	1.83	78	23.29	30	male
6	1.75	70	22.86	32	male
7	1.79	75	23.41	53	male
8	1.74	63	20.81	20	male
9	1.70	68	23.53	24	male
10	1.82	73	22.04	30	male
11	1.74	81	26.75	31	male
12	1.73	67	22.39	19	male
13	1.71	60	20.52	23	male
14	1.68	66	23.38	21	female
15	1.88	75	21.22	20	male
16	1.83	82	24.49	28	male

**Table 2:** Optimised hyperparameters.

Hyperparameter	Description	Optimised Value
*filter_size*	The size of filters in the convolutional layer	3
*filters_num*	The number of filters in the convolutional layer	189
*mini_bs*	The number of training samples used in a single iteration	16
*feature_num*	The number of neurons in the first fully connected layer (FC1)	11
*lr*	The initial learning rate	9.708 × 10^−3^
*epoch_num*	The number of training epochs	39
*L2_coe*	L2 regularisation coefficient	0.098

**Table 3:** Mean tidal volume of the individual subjects and mean absolute errors and relative errors of the tidal volumes obtained using the two approaches, refers to the tidal volumes measured using the spirometer.

Subject	Mean *V_T,Spiro_* mL	Regression Mean abs. Error mL	Regression Mean rel. Error %	CNN Approach Mean abs. Error mL	CNN Approach Mean rel. Error %
1	906	73.4	8	389.8	43
2	689	92.2	13	370.5	54
3	953	168.8	18	157.6	17
4	626	168.3	27	244.4	39
5	976	50.7	5	76.3	8
6	1030	339.6	33	118.4	11
7	1637	115.1	7	579.8	35
8	608	100.7	17	275.8	45
9	987	62.4	6	245.8	25
10	985	70.7	7	224.8	23
11	757	66.1	9	130.6	17
12	559	40.2	7	234.3	42
13	985	55.5	6	299.9	30
14	714	58.4	8	306.9	43
15	992	80.0	8	110.0	11
16	918	34.0	4	97.5	11

The mean absolute error of the regression approach was 98 mL, while the mean absolute error of the CNN approach was 241 mL.

## Data Availability

The data presented in this study are available upon request from the corresponding author.

## Abbreviations

The following abbreviations are used in this manuscript:

BN layer	Batch normalisation layer
CNN	Convolutional Neural Network
ECG	Electrocardiogram
FC layer	Fully connected layer
IMU	Inertial Measurement Unit
LSTM	Neural Network with Long Short-Term Memory Architecture
MoCap	Motion Capture System
NN	Neural Network
OEP	Optoelectronic Plethysmography
ReLu layer	Rectified linear unit layer
RIP	Respiratory Inductance Plethysmography
RMSE	Root mean squared error
RR	Respiratory Rate
*V_T,Spiro_*	Tidal Volume, obtained via Spirometer
*V_T,Reg_*	Tidal Volume, obtained via Regression
*V_T,CNN_*	Tidal Volume, obtained using the Convolutional Neural Network
